# DYSKIMOT: An Ultra-Low-Cost Inertial Sensor to Assess Head’s Rotational Kinematics in Adults during the Didren-Laser Test

**DOI:** 10.3390/s20030833

**Published:** 2020-02-04

**Authors:** Renaud Hage, Christine Detrembleur, Frédéric Dierick, Laurent Pitance, Laurent Jojczyk, Wesley Estievenart, Fabien Buisseret

**Affiliations:** 1 Laboratoire NMSK, Institut de Recherche Expérimentale et Clinique, Université Catholique de Louvain, 1200 Brussels, Belgium; christine.detrembleur@uclouvain.be (C.D.); laurent.pitance@uclouvain.be (L.P.); 2 CeREF, Chaussée de Binche 159, 7000 Mons, Belgium; frederic.dierick@gmail.com (F.D.); jojczykl@helha.be (L.J.); estievenartw@helha.be (W.E.); buisseretf@helha.be (F.B.); 3 Centre National de Rééducation Fonctionnelle et de Réadaptation—Rehazenter, Laboratoire d’Analyse du Mouvement et de la Posture (LAMP), 2674 Luxembourg, Luxembourg; 4 Service de Physique Nucléaire et Subnucléaire, UMONS, Research Institute for Complex Systems, 20 Place du Parc, 7000 Mons, Belgium

**Keywords:** inertial sensor, kinematics, head rotation, ecological research

## Abstract

Various noninvasive measurement devices can be used to assess cervical motion. The size, complexity, and cost of gold-standard systems make them not suited to clinical practice, and actually difficult to use outside a dedicated laboratory. Nowadays, ultra-low-cost inertial measurement units are available, but without any packaging or a user-friendly interface. The so-called DYSKIMOT is a home-designed, small-sized, motion sensor based on the latter technology, aiming at being used by clinicians in “real-life situations”. DYSKIMOT was compared with a gold-standard optoelectronic system (Elite). Our goal was to evaluate the DYSKIMOT accuracy in assessing fast head rotations kinematics. Kinematics was simultaneously recorded by systems during the execution of the DidRen Laser test and performed by 15 participants and nine patients. Kinematic variables were computed from the position, speed and acceleration time series. Two-way ANOVA, Passing–Bablok regressions, and dynamic time warping analysis showed good to excellent agreement between Elite and DYSKIMOT, both at the qualitative level of the time series shape and at the quantitative level of peculiar kinematical events’ measured values. In conclusion, DYSKIMOT sensor is as relevant as a gold-standard system to assess kinematical features during fast head rotations in participants and patients, demonstrating its usefulness in both clinical practice and research environments.

## 1. Introduction

Neck pain is a common neuromusculoskeletal symptom with a prevalence ranging from 22% to 70%, increasing with age and affecting most often women around 50 years old [1]. It is the fourth leading cause of years lived with disability in 188 countries during the period 1990–2013 [2]. Therefore, the correct identification of the source of neck pain is paramount. However, probably due to imperfect diagnosis, the majority of patients with neck pain are still nowadays called “non-specific” [3].

According to the Bayesian inference, a medical diagnosis indicates that one disorder (e.g., muscular, discogenic, lack of sensorimotor control deficits) more than another is probably the cause of a patient’s symptoms, and thus, investigations are needed to reinforce or refute the hypothetical diagnosis [4]. In accordance with the literature [2,5,6,7], diagnoses and therapeutic interventions for neck pain should be informed using quantitative (strength and range of motion) and qualitative (sensorimotor appraisal) assessment of neck rotation. Quantitative devices have been reported to be superior to visual estimation to assess the cervical range of motion [8], the most popular method used by clinicians being goniometry [9]. Although very easy to use, goniometry has a margin of error of about 5° [9]. Moreover, maybe more important than movement amplitude [5,10], the evaluation of sensorimotor function has demonstrated its importance in developing a better understanding of the pathophysiological mechanisms associated with cervical pain [6] both in cases of specific neck pain such as traumatic neck pain [11], as well as for idiopathic neck pain [12]. Therefore, in an attempt to better define the clinical picture of patients by focusing on head movement [5,13,14] especially in axial rotation [15], clinicians show increased interest in quantitative devices that can accurately monitor movement.

Various noninvasive three-dimensional motion capture systems are used in the field of cervical research in order to evaluate kinematic variables going beyond simple range of motion such as speed, acceleration and deceleration using electrogoniometers [16], ultrasound waves [17], optical-based systems [18,19] and inertial sensors [20] and so on. Nevertheless, their dimension, complexity, and cost make such systems often difficult to use in clinical practice. The need for compact, user-friendly and low-cost measurement devices that can bring relevant information in everyday clinical practice is therefore obvious and goes beyond neck exploration, although we chose to focus on that topic in the present study.

Inertial measurement units sensors (IMUs) began to be applied to human movement before 2000 [21]. IMUs consist of accelerometers and gyroscopes which are organized in orthogonal triads in order to obtain three-dimensional kinematics [22]. Most often, IMUs are now supplemented by magnetometers and thermometers and are called MARG sensors (magnetic angular rate and gravity sensors). This technology has the advantage of not requiring external equipment such as cameras to acquire the orientation and position of the human segments, and it does not limit the subject’s movement to the volume covered by the cameras. IMUs or MARGs thus seem to be the appropriate basic tool to design a device which could be easily used in a clinical and ecological environment [23,24]. Note that this technology suffers from high measurement noise and drift [25] that can mostly be cured by a Kalman filter [25]. Nowadays, the large-scale production MARGs sensors make them affordable compared to gold-standard systems but nevertheless the prices are several thousand euros (e.g., Vicon^®^, XSENS^®^). Other MARGs may indeed be bought at typical prices less than 50 €, still without any packaging nor user-friendly interface.

It is in this context that our team designed a small-sized, light, and ultra-low-cost inertial sensor called DYSKIMOT. After first laboratory tests, our goal was to evaluate the accuracy of DYSKIMOT compared to a gold-standard optoelectronic system when performing a clinical sensorimotor test developed by Hage et al. (i.e., the DidRen-Laser Test) in small groups of asymptomatic and symptomatic neck pain participants [26]. We selected different dynamic outcomes to evaluate our DYSKIMOT [27,28]: range of motion, peak speed, average speed, peak acceleration, and peak deceleration.

## 2. Materials and Methods

### 2.1. Participants

Fifteen cervical non-disabled participants (NDP) (3 females, 12 males) and 9 cervical disabled patients (DP) (4 females, 5 males) were recruited from students in University hospital and among researchers’ patients to participate in this study, see Table 1. Inclusion criteria for NDP were the absence of neck pain episodes in the last 6 months and a neck disability index (NDI) [29] score of less than or equal to 8%. Inclusion criteria for the DP were a numeric pain rating scale (NPRS) equal to or greater than 3/10 [30] and an NDI > 8%. Exclusion criteria were for NDP and DP: impaired cognition, blindness, deafness, dizziness, or vestibular disorders diagnosed by a physician. Participants and patients did not exhibit any neuromusculoskeletal or neurologic disorder that could influence the performance of head rotation in the horizontal plane. The participants signed informed consent and gave permission to publish their case details. The study was approved by the local ethics committee (Comité d’Ethique Hospitalo-Facultaire Saint-Luc-UCL (IRB 00001530)) and conducted in accordance with the declaration of Helsinki.

### 2.2. The DidRen Laser Test

The DidRen Laser Test [26] was used to assess neck mobility through standardized axial rotations of the head in NDP and DP.

**Figure 1:**
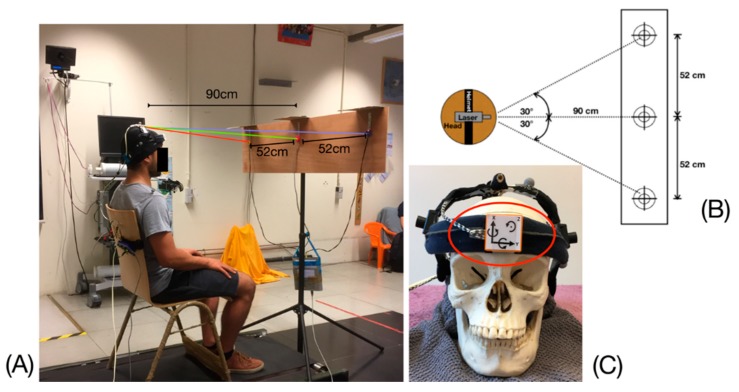
(**A**) DidRen Laser Test installation device. (**B**) Schematic view from above. The passage from one target to another induces an axial rotation of the head of 30° either to the left or to the right sides of the bodyline. (**C**) The Helmet worn by the participant with the Laser on the top. The DYSKIMOT sensor can be seen (red circle) at the front of the helmet.

After watching an explanatory video, participants sat on a chair with backrest, without armrests, placed at 90 cm from a vertical panel equipped with 3 targets (LEDs) arranged horizontally and located 52 cm apart (Figure 1). Participants wore an adjustable helmet with a laser beam attached on the top was worn by the participant (Figure 1). The experimenter (RH) adjusted the helmet so that the laser hit the central target while the participant was in a neutral position before the test began. The instructions were the same for all participants: “You must reach the targets as fast as you can and perform the head movement without moving your shoulders”. The targets were then turned on in a predefined sequence and the participant’s task was to rotate his/her head so that the laser beam hit the target as quickly as possible. When the laser beam was stabilized by the participant on a target for at least 0.5 s, the target LED lit up and an audible signal was emitted. A complete test was composed of 5 cycles of cervical axial rotation to the right and left sides respectively.

A first test was carried out to familiarize the participant and a second for data recording and analyses [26].

### 2.3. Motion Sensors

#### 2.3.1. Elite System (BTS)

**Figure 2:**
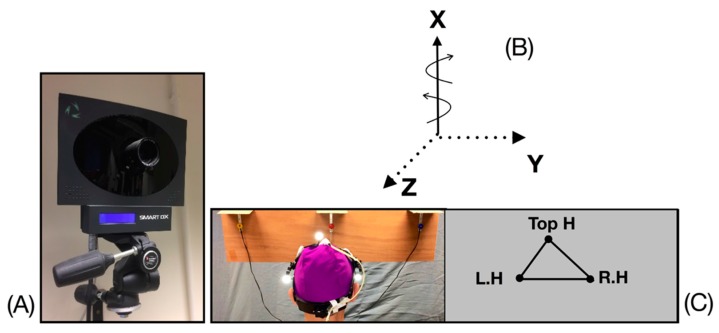
(**A**) Infra-red cameras (ELITE, BTS, Milan, Italy). (**B**) Head axis of rotation is denoted as X. (**C**) Placement of the reflective markers on the head.

An optoelectronic system composed of 8 infra-red cameras (ELITE, BTS, Milan, Italy) (Figure 2A) with sampling frequency of $\bar{f}$ = 200 Hz test carried out the three-dimensional recording of the markers on the helmet during the DidRen Laser. A kinematic model composed of 3 markers on a helmet and fixed during all experimentations representing the head was used and adapted from [31] (Figure 2B,C). Helmet markers were positioned such that one was just aside the top of the head (Top H) and positioned next to the laser, and two on each side of the head (R.H and L.H) (Figure 2C). Real time detection of head rotation markers was executed around a coordinate system such that the axis of rotation for head axial rotations was X (inferior-superior axis). The Y-axis was aligned with participant’s mediolateral axis at the beginning of the test and the Z-axis was aligned with the antero-posterior axis. This is illustrated in Figure 2B. The system was previously calibrated within the infra-red camera’s field of view [31] and the instantaneous X, Y, and Z coordinates of the three markers were recorded, leading to ${\vec{X}}_{Top\;H}$, ${\vec{X}}_{L.H},$ and ${\vec{X}}_{R.H}$. The vector $\vec{u}={\vec{X}}_{Top\;H}-\frac{{\vec{X}}_{L.H}+{\vec{X}}_{RH}}{2}$ gives the orientation of antero-posterior axis (coinciding with that of the laser beam).

The angular displacement time series of the head, ${\bar{\theta }}_{i}$, has been computed from the coordinates of the markers as described in details in [32]: ${\bar{\theta }}_{i}=cos^{-1}\left(\frac{{\vec{u}}_{i}.\;{\vec{u}}_{0}}{\left||{\vec{u}}_{i}|\right|\left||{\vec{u}}_{0}|\right|}\right)$. The index *i* denotes the vector at time *i* $\Delta \bar{t},\;\Delta \bar{t}$ = 1/$\bar{f}$. The angular velocity was then computed as ${\bar{\omega }}_{i}=\frac{{\bar{\theta }}_{i+n}-{\bar{\theta }}_{i-n}}{2.n.\Delta \bar{t}\;}$ with *n* = 5 and, similarly, the angular acceleration has been computed as ${\bar{\alpha }}_{i}=\frac{{\bar{\omega }}_{i+n}-{\bar{\omega }}_{i-n}}{2.n.\Delta \bar{t}\;}$. The choice *n* = 5 guaranteed an optimal smoothness of the curves both for Elite and DYSKIMOT time series (assessed by visual inspection).

#### 2.3.2. DYSKIMOT

**Figure 3:**
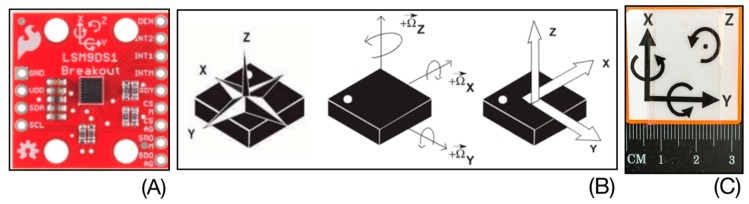
(**A**) Micro Electro-Mechanical Systems (MEMS). (**B**) 3-axis accelerometer, a 3-axis gyrometer, a 3-axis magnetometer and a temperature sensor. (**C**) Dimension of the DYSKIMOT.

The DYSKIMOT sensor is a MARG sensor based on the Micro-Electro-Mechanical Systems (MEMS) IMU LSM9DS1 (SparkFun, 14 €), with a mass of 10.44 gr and size of 3 × 3 cm (Figure 3A,C). It is composed of 3-axis accelerometer, gyrometer and magnetometer, plus a temperature sensor (Figure 3B). These internal components respectively measure acceleration (in [g], ±16 [g]), angular velocity (in [°/s], ±2000 [°/s]) and magnetic field (in [gauss], ±16 [gauss]).The apparatus can operate between −40 °C and +85 °C. The sensitivity depends on the sensor and on the selected range; detailed information is given in the datasheet (https://www.st.com/en/mems-and-sensors/lsm9ds1.html). For example, the gyrometer sensitivity is 8.75 10^−3^ °/s /LSB at the range ± 245 °/s, i.e., the range we use in the present study. Communication with other electronic components is made via serial peripheral interface bus (SPI) or inter-integrated circuit (I2C) protocol. The data recorded at a sampling frequency *f* = 100 Hz are transmitted to a PC via an Arduino Uno Rev 3 (23 €) and a USB cable (RS232 serial link). That sampling frequency was actually the maximal reachable with the devices used. The Arduino contains the data recovery program, using the SparkFun library provided for this sensor, and transfers them to a home-made acquisition software.

The DYSKIMOT sensor was placed in front of the helmet (Figure 1C) with the X-axis in the vertical direction (inferior-superior axis). The Y-axis was aligned with participant’s mediolateral axis at the beginning of the test and the Z-axis was aligned with the antero-posterior axis. This choice has two advantages. From a clinical point of view it is the most reliable position to record cervical axial rotation as shown in [33]. From an algorithmic point a view, the sensor orientation is such that the relevant information about the DidRen Test is fully contained in the X-component of angular velocity measured by the gyroscope. The latter time series was denoted $\omega_{i}$. A trapezoidal integration gave the head’s rotation angles $\theta_{i}$, where the constant of integration was chosen such that the angle was zero at the beginning of the test. The derivative $\alpha_{i}=\frac{\omega_{i+n}-\omega_{i-n}}{2.n.\Delta t\;}$ with *n* = 5 and Δ*t* = 1*/f* provided the head’s angular acceleration. Angles computed from the gyroscope showed a linear drift. Since the DidRen Laser Test consists of quasi-periodic rotations of 30° around a neutral position, a straightforward way of removing the drift is to subtract the least square regression line from the time series $\theta_{i}$. Notice that Elite (DYSKIMOT) time series are written with (without) a bar.

**Figure 4:**
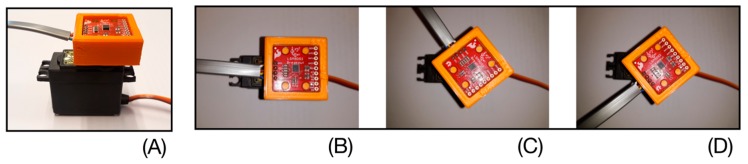
(**A**) Servo motor + housing adapted to its axis to fix the MARG. (**B**) Angle = 0°, (**C**) Angle = +30°, (**D**) Angle = −30°.

**Figure 5:**
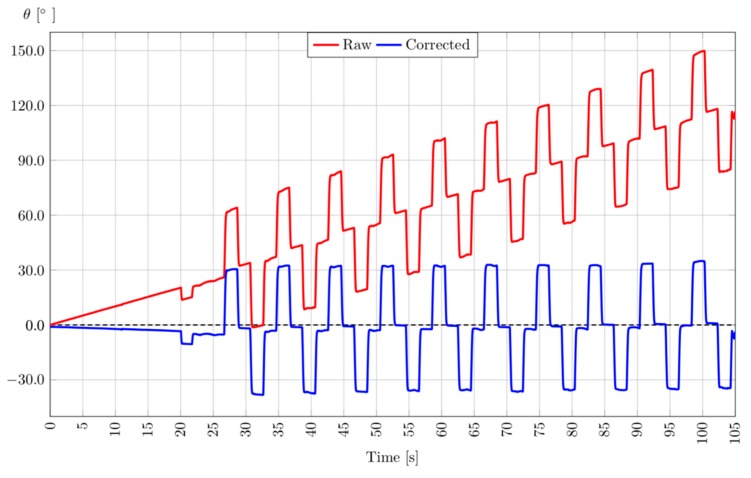
Example of linear drift due to integration of DYSKIMOT’s raw angular velocity (red line) and correction of the drift of the test angle Z with the servo motor (blue line). The corrected angle (blue line) is obtained by subtraction of the regression line to the raw angle. This plot has been obtained by fixing the DYSKIMOT sensor on a servo motor (MG995, Tower Pro) performing successive and opposite rotations of amplitude 30°.

Before using the DYSKIMOT in this study, a test was performed using a sensor attached to a servo motor (see Figure 4) to mimic the sequence of the cervical axial rotation during the DidRen test, i.e., angles from 30° to the left and right by going back through the 0 angle (Figure 4). The servo motor with an Arduino Uno Rev 3, was programmed to perform the sequence repeatedly. The result can be seen in Figure 5. The sensor was kept static during the first 20 s of the test. The linear drift is clearly observable on the raw angular data and the parameters of this line are computed by a least squares regression. Then comes the activation of the actuator and the beginning of the sequence (around 25 s) started. The fitted linear drift was eventually subtracted from the raw angular data. Such a procedure is satisfactory for time series displaying the typical behavior of the DidRen Laser Test (Figure 5). Such a procedure may actually work in all cases, including non-periodic tests. The regression line parameters may even be stored provided they do not change over time or with temperature. We checked that the drift stays linear at larger time scales (30 min).

### 2.4. Data Analysis

Signals from DYSKIMOT and Elite were synchronized by an external digital trigger (National Instrument, Austin TX, United States). Since the frequencies of both sensors were different (100 Hz vs 200 Hz), the accuracy of the synchronisation of the time series $\bar{\theta },\;\bar{\omega },\;\bar{\alpha }$ (Elite) and θ, ω, α (DYSKIMOT) is in the order of 5 ms.

**Figure 6:**
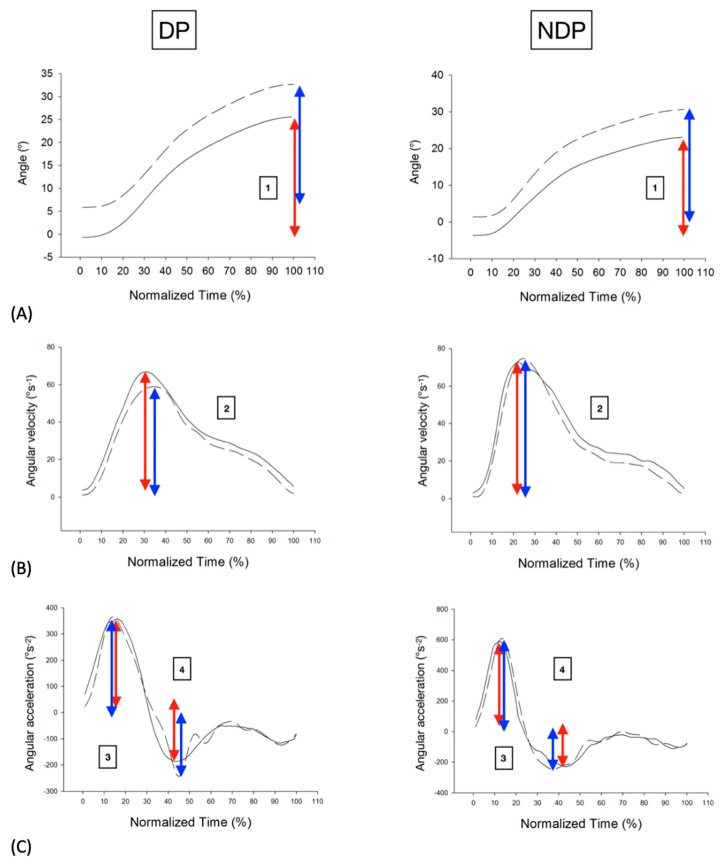
Typical plots of variables analyzed during one right rotation in a DP (34 years, Male, NDI = 22, NRPS = 5) and an NDP (25 years, Male, NDI = 0, NPRS = 0): (**A**) Angle; (**B**) Angular velocity; (**C**) Angular acceleration. Elite curves (dotted lines) can be compared to DYSKIMOT ones (solid lines). Computed parameters are illustrated by blue (Elite) or red (DYSKIMOT) arrows. (1) angle; (2) peak angular velocity; (3) peak angular acceleration; (4) peak angular deceleration.

**Figure 7:**
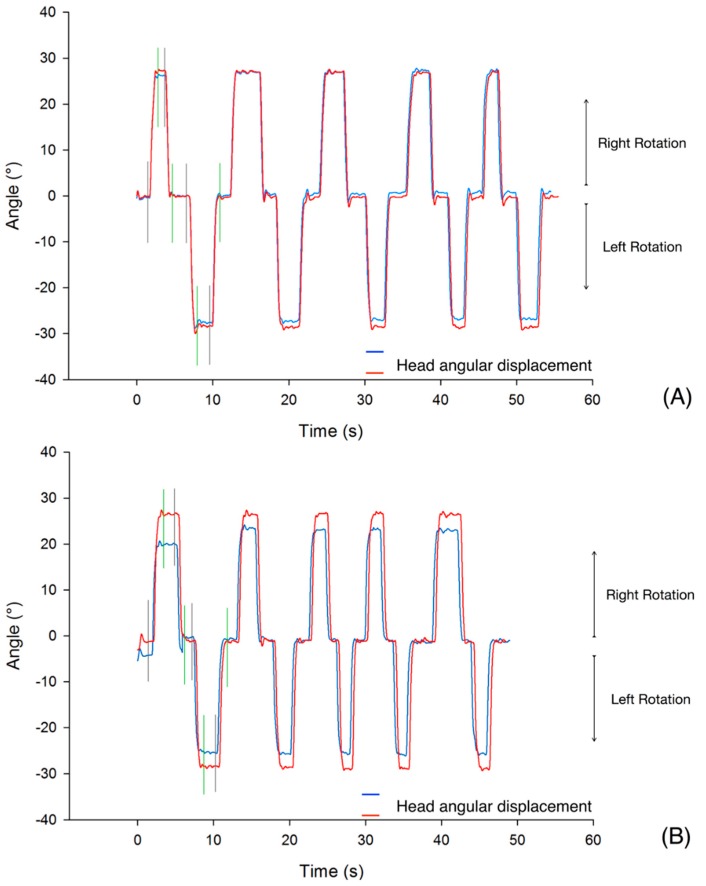
Typical traces of the head motion during the 5 cycles of the DidRen Laser Test showing comparison of Elite (red line) and DYSKIMOT (Blue line) angle discrepancies. In (**A**), the best angle agreement between Elite and DYSKIMOT (difference = 0.6°, mean angle during 5 cycles = 25.7°) in an DP (34 years, male, NDI=22, NPRS = 5), and in (**B**) the worst agreement (difference = 4.0°, mean angle during 5 cycles = 27.5°) in a NDP (22 years, male, NDI = 0, NPRS = 0). Cursors indicating the beginning (grey) and end (green) of one axial rotation movement are shown.

Then the following parameters were calculated during each cycle and averaged on the 5 cycles achieved by each participant, see Figure 6 and Figure 7 for a graphical illustration of our computational procedure: (1) angle (range of motion, in °); (2) peak angular velocity (maximum angular velocity reached, in °s^−1^); average angular velocity (in °s^−1^); (3) peak angular acceleration (maximum angular acceleration reached, in °s^−2^); (4) peak angular deceleration (minimum angular acceleration reached, in °s^−2^). The beginning of all cycles has been manually marked by one of the authors (RH) within a homemade software that performed the averages over the 5 cycles for each trial. The peak value of a given time series X_i_ has been computed to be max(X_i_) unless the maximal value was judged to be an artefact by visual inspection of the curves. Then, the value below this maximum was retained.

Although our goal was to measure the agreement between Elite and DYSKIMOT sensors for ND and NDP participants, the computed parameters were of clinical interest, as neck velocity during fast rotation can discriminate between nonspecific neck pain and healthy control [13,14].

A Passing–Bablok regression [34], which allows to compare the DYSKIMOT vs Elite data, was performed on the individual values of the parameters for DP and NDP simultaneously so that the agreement between both sensors could be appraised and summarized by a “calibration line”.

A two-way ANOVA was then used to assess potential differences between the two systems (System factor: Elite or DYSKIMOT) and between the groups (Status factor: NDP or DP) for the parameters mentioned above. When the ANOVA indicated significant interaction, a post hoc Holm-Sidak analysis with pairwise multiple comparisons was carried out. Significance was fixed at *p* < 0.05 and all statistical procedures were performed with SigmaPlot 13 (Systat Software, Inc).

Finally, a dynamic time warping (DTW) analysis (without windowing) was carried out on the z-normalized data and the Euclidian DTW distances between the time series angle $\left(\theta ,\bar{\theta }\right)$, angular velocity $\left(\omega ,\bar{\omega }\right)$, angular acceleration ($\alpha ,\bar{\alpha })$ for DYSKIMOT and Elite were calculated for all the participants and then averaged. The z-normalization consisted in replacing a time series *X* by $\frac{X-E\left(X\right)}{SD\left(X\right)}$, *E* and *SD* denoting the average and standard deviation respectively.

The Passing–Bablok regressions and the DTW were performed by using R v3.4.2 and the packages mcr and dtw.

It is worth saying that the accuracy of synchronization is not a matter of concern: the parameters have been independently computed from Elite and DYSKIMOT time series, and no locality constraint has been added in the DTW procedure through a window parameter. Synchronization was mainly a facilitating tool for graphical exploration of the data.

## 3. Results

A total of twenty-four participants were recruited with their demographics charateristics detailed in Table 1.

Results of the two-way ANOVA are shown in Table 2. A statistically significant difference was observed in the angle (*p* < 0.001) and average angular velocity (*p* < 0.022) for the System factor, i.e., Elite and DYSKIMOT lead to different means. Neither the status factor nor the interaction effects lead to statistically significant differences.

**Figure 8:**
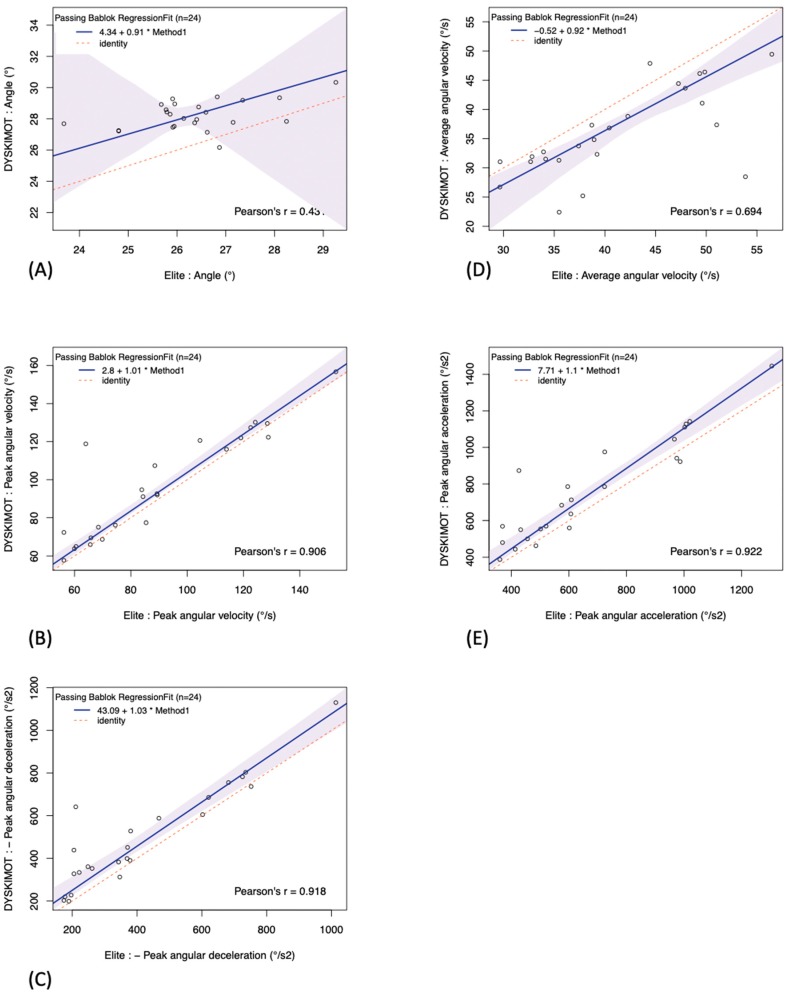
Passing-Bablok regressions showing the individual parameters computed from the DYSKIMOT and Elite data (points): (**A**) Angle, (**B**) Peak angular velocity, (**C**) Peak angular deceleration, (**D**) Average angular velocity, (**E**) Peak angular acceleration. The regression line (solid line) is given and compared to the identity line (dotted line). The 95% confidence interval for the linear fit is also displayed (colored band).

The results of Passing–Bablok regressions are shown in Table 3. The slopes were close to 1, with the best agreement observed for the peak angular velocity, and Pearson’s coefficients range from 0.431 to 0.922, i.e., there is a moderate to excellent linear correlation between DYSKIMOT and Elite results. The agreement between both systems can be graphically appraised in Figure 8. Angles show the poorest linear correlation, resulting in a large uncertainty in the best fit (large 95% confidence interval). The other parameters show better linear correlation, and the best fit is known with better accuracy (smaller 95% confidence intervals).

**Figure 9:**
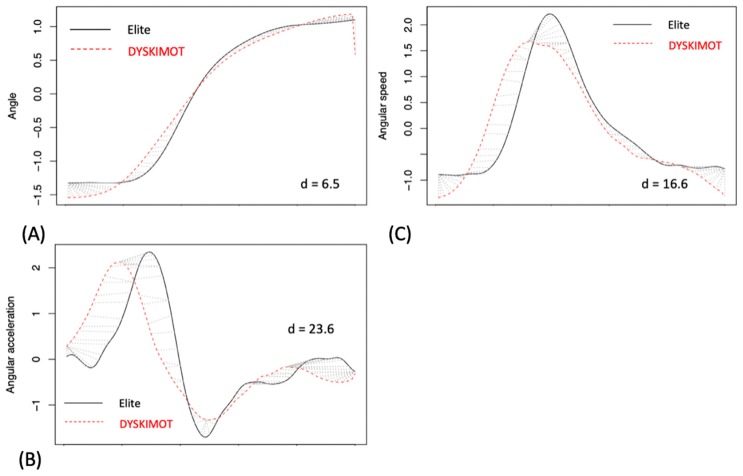
Typical plots of DTW matching between Elite (black solid lines) and DYSKIMOT (red dashed lines) analyzed for z-normalized data in one NDP (22 years, Male, NDI = 0, NRPS = 0) participant: (**A**) Angle, (**B**) Angular acceleration, (**C**) Angular speed. The DTW distance is added for completeness.

The DTW distance (d) allows for an estimation of the closeness of DYSKIMOT and Elite systems for the whole time series, not only for selected valued. We obtained 5.16 ± 2.68, 8.82 ± 5.80, and 14.40 ± 7.14 for angle, angular velocity, and angular acceleration time series respectively. Typical traces are shown in Figure 9.

## 4. Discussion

Before comparing Elite and DYKIMOT measurements we recall their main features in Table 4.

The two-way ANOVA revealed that angle and average angular velocity were significantly different between Elite and DYSKIMOT systems. The difference in angle (1.76°) between the two systems is lower than the standard clinical angle evaluation of 5° reported via classical goniometry [9]. Such difference between the two systems is not clinically relevant, as an error of 2° is acceptable in most clinical situations [35]. Concerning the average angular velocity, the difference of −5.50 °s^−1^ may come from the errors induced by the derivation of the Elite position. This result is lower than the difference necessary to detect significant differences (7.1 °s^−1^) between adults and children [28]. Nevertheless, the clinical significance of a such difference is currently unknown. Apart from these differences, two other ANOVA results may be noted. First, as in [36], no signifiant difference between DP and NDP were observed for all variables studied. Second, the interactions effects (System x Status) did not induce significant differences. At this stage, DYSKIMOT and Elite give broadly similar results, but the computed parameters do not allow to distinguish between DP and NDP, at least in our population. Another point preventing the separation of DP and NDP is that the differences of the means were generally larger for the system factor than for the status factor: The accuracy of the DYSKIMOT device has to be improved, e.g., by appropriate filtering of the raw data and a better integration of gyrometer data, to reduce these discrepancies and improve the diagnostic ability of the sensor.

In a classic way [37,38,39], we have previously used Bland and Altman’s method to evaluate the agreement between DYSKIMOT and Elite [40]. It appeared that the Bland and Altman’s plots did not show a trend with the mean values of the measurements. The Bland and Altman plot for the angle parameter showed more points outside or close to the limits of agreement than the other plots, which is an indication that agreement between both systems is less obvious for the range of motion than for other parameters [40]. Since this method does not provide any quantified results on the comparison and leaves the user to decide whether this agreement is clinically acceptable or not, we analyzed the agreement between DYSKIMOT and Elite using Passing–Bablok regressions. The Passing–Bablok regression method is a non-parametric method for estimating the slope and the intercept of the linear relationship between two compared [34]. These two parameters are valued by medians and are less sensitive to extreme data and not making assumptions about errors distribution [41]. In our results, the Passing–Bablok indicated that the link between same parameters computed from both systems was well compatible with a linear shape (*r =* 0.694 to 0.922) for all parameters but angle, for which Pearson’s coefficient was rather weak (*r =* 0.431) [42]. Nonzero offsets were observed but the 95% confidence intervals were large and always contained 0 value, while the slopes were close to 1 (up to 10% accuracy) with 95% confidence intervals always containing the value 1. Another advantage to this method is that by assuming that Elite results are gold-standard values, the Passing–Bablok regressions could be used to convert measured parameters with DYSKIMOT into “exact values” which are the Elite ones.

Although DTW has been known in the field of acoustic signal comparison [43], it has also been proposed for the purposes of similarity analysis during the functional pattern of gait [44], but never to compare motion neck signals obtained by two different devices. DTW is, by definition, sensitive for measuring two sequences with different lengths using dynamic programming [45]. In this work, the DTW distance between Elite and DYSKIMOT curves was adopted as an indicator of the similarity (up to an affine transformation) between the curves. In other words, the question was: Do both systems measures the same qualitative behaviors in position, angular velocity, and angular acceleration? Although angle measurements displayed a poor agreement between both systems, the DTW distance between DYSKIMOT and Elite angle was minimal: This result was expected since the structure of angle was simpler than angular velocity and angular acceleration. The DTW distance then increases between the angular velocity and the angular acceleration of the DYSKIMOT and Elite systems. This mostly results in the noise induced by the successive derivations, showing that qualitative features of these curves, especially the angular acceleration, should be interpreted carefully and might be artefacts of the sensor used.

The identification of particular kinematic events is relevant for the clinical assessment of patients, but the global shape of time series may contain more information of clinical interest. In our case for example, it is known that patients with neck pain have poorer sensory-motor control with open eyes, characterized by an increase in joint positioning error and a decrease in speed and acceleration during all movements [12]. The absence of difference in our kinematics data between patients and participants could seem unexpected as previous studies showed significant differences in terms of kinematics [5,46]. However this absence of difference could be explained by our sample size, resulted in low power, and by the difficulty for the DidRen laser to discriminate between such groups [47,48].

An obvious limitation of the present study is that we restricted our comparison of Elite and DYSKIMOT to cervical movements, while potential clinical applications may involve any other joints. Another limitation is that we used “naïve” drift correction following data acquisitions, which had to be implemented in real-time in the software. The Arduino prevented us to reach the desired frequency of 100 Hz with real-time complex filters like Kalman or Mahony. A future development would be the replacement of the Arduino by a slightly more expensive controller (ARM, 30 €), that will allow for real-time filtering and eventually for real-time angular data visualization without entailing too much the low-cost aspect of the DYSKIMOT project. It is therefore obvious that the presented experiments were carried out with a non-user-friendly interface, particularly because of the drift-related problems. However, as the goal of this study was to evaluate the accuracy of a device that could be used by clinicians in clinical practice, we have chosen to leave this concern for future works. A user-friendly interface is currently under development.

In conclusion, the DYSKIMOT-based analysis system compares fairly well to a gold-standard optoelectronic system (Elite) up to linear errors. This ultra-low-cost sensor is recommended for clinical use as it provides more accurate information than the commonly used systems in clinical practice.

## Figures and Tables

**Table 1:** Characteristics of the participants in NDP and DP groups. Data are given either under mean ± SD or median [Q1–Q3] form.

	NDP (n = 15)	DP (n = 9)
Age (years)	24 ± 3	31 ± 14
Gender (men/women)	12/3	5/4
BMI (kg/m^2^)	22.2 ± 2.7	21.8 ± 2.3
NDI (%)	0 [0–0]	14 [10–16]
NPRS (/10)	0 [0–0]	3 [0–0] ^1^

^1^ SD = Standard Deviation, BMI = Body Mass Index, Q1 = First Quartile, Q3 = Third Quartile, NDI = Neck Disability Index. NPRS = Numeric Pain Rating Scale, NDP = Non-Disabled Participants. DP = disabled participants.

**Table 2:** Results of the two-way ANOVA performed on the parameters. *p* values are given for the differences between Elite and DYSKIMOT (System), between DP and NDP (Status) and for the interaction effect System x Status. *p* values lower than 0.05 are given in bold font.

	ANOVA	Difference of the Means (Dyskimot-Elite or DP-NDP)	*p*
Angle (°)	System	1.76	**<0.001**
	Status	−0.398	0.157
	System x Status		0.094
Average angular velocity (°s^−1^)	System	−5.50	**0.022**
	Status	1.72	0.462
	System x Status		0.655
Peak angular velocity (°s^−1^)	System	5.74	0.498
	Status	6.22	0.630
	System x Status		0.708
Peak angular acceleration (°s^−2^)	System	89.9	0.282
	Status	59.8	0.473
	System x Status		0.880
Peak angular deceleration (°s^−2^)	System	−81.3	0.261
	Status	−46.6	0.517
	System x Status		0.955

**Table 3:** Results of Passing–Bablok regressions performed on the computed parameters. Slope and Offset are given with their 95% confidence intervals (between brackets).

	Slope	Offset	r
Angle (°)	0.908 [−2.09, 1.86]	4.34 [−20.6, 82.5]	0.431
Average angular velocity (°s^−^^1^)	0.922 [0.713, 1.32]	−0.518 [−18.0, 7.57]	0.694
Peak angular velocity (°s^−1^)	1.01 [0.942, 1.11]	2.80 [−5.27, 10.2]	0.906
Peak angular acceleration (°s^−2^)	1.10 [0.939, 1.20]	7.71 [−50.4, 125]	0.922
Peak angular deceleration (°s^−^^2^)	1.04 [0.906, 1.13]	43.1 [−0.623, 131]	0.918

**Table 4:** Summary of Elite and DYSKIMOT main features relative to the present study.

Elite		DYSKIMOT	
Infrared digital cameras	8	MARG sensor	IMU LSM9DS1
Resolution	1.5 Mpixel	Gyrometer range	±245 °/s
Sample frequency	200 Hz	Sample frequency	100 Hz
Accuracy/volume	<0.1 mm on 4 × 3 × 3 m	Gyrometer sensitivity	8.75 10^−3^ °/s
